# Cefoselis enhances breast cancer chemosensitivity by directly targeting GRP78/LRP5 signalling of cancer stem cells

**DOI:** 10.1002/ctm2.1119

**Published:** 2023-02-19

**Authors:** Yifeng Zheng, Neng Wang, Shengqi Wang, Bo Pan, Bowen Yang, Juping Zhang, Xuan Wang, Zhiyu Wang

**Affiliations:** ^1^ Integrative Research Laboratory of Breast Cancer Discipline of Integrated Chinese and Western Medicine The Second Clinical College of Guangzhou University of Chinese Medicine Guangzhou Guangdong China; ^2^ State Key Laboratory of Dampness Syndrome of Chinese Medicine The Second Affiliated Hospital of Guangzhou University of Chinese Medicine Guangzhou China; ^3^ Guangdong Provincial Key Laboratory of Clinical Research on Traditional Chinese Medicine Syndrome Guangdong Provincial Academy of Chinese Medical Sciences Guangdong Provincial Hospital of Chinese Medicine Guangzhou Guangdong China; ^4^ Guangdong‐Hong Kong‐Macau Joint Lab on Chinese Medicine and Immune Disease Research Guangzhou University of Chinese Medicine Guangzhou Guangdong China; ^5^ The Research Center for Integrative Medicine School of Basic Medical Sciences Guangzhou University of Chinese Medicine Guangzhou Guangdong China

Dear editor,

Stress‐induced cellular defence machinery is significant for regulating breast cancer stem cells (CSCs).^1^, ^2^, ^3^ GRP78 is an endoplasmic reticulum (ER) stress protein and has been reported to be overexpressed in multiple malignancies.^4^ In this study, we shed novel light on the role of GRP78 in regulating breast CSCs via LRP5/β‐catenin signalling. Cefoselis is a widely used β‐lactam antibiotic with high efficacy and safety.^5^ Here, we identified cefoselis as a GRP78‐targeting agent for eliminating breast CSCs.

We first evaluate the clinical implications of GRP78 expression in breast cancer and its potential as a druggable target. Bioinformatics analysis indicated that *GRP78* levels were higher in tumours than in para‐tumour tissues. The overall survival of GRP78^high^ breast cancer patients was significantly poorer compared to GRP78^low^ patients (Figure 1A). Moreover, GRP78 expression was significantly higher in CD44^+^/CD24^−^ or ALDH^+^ breast CSCs (Figure 1B). A tissue microarray analysis (*n* = 118) confirmed that GRP78 was highly correlated with prognosis and CSC‐related signalling in breast cancer (Figure S1, Tables S1 and S2). Similarly, GRP78 expression was remarkably upregulated in breast cancer stem‐like cells (Figure 1C). Notably, GRP78 was highly expressed on the cell surface of breast cancer stem‐like cells but was reduced following differentiation (Figure 1D–E). Previous studies have indicated that GRP78 shifts to the cell membrane under ER stress.^6^, ^7^, ^8^ Herein, it was also observed that paclitaxel treatment induced GRP78 translocation to the cell surface (Figure 1F). GRP78 expression and the stem‐like cell population were significantly elevated in paclitaxel‐resistant cells (Figure S2). GRP78 knockdown resulted in a significant reduction of breast cancer stem‐like cell numbers, mammosphere formation abilities, and β‐catenin nuclear localisation in both breast cancer cell lines, whereas GRP78 overexpression presented the opposite effects (Figures 1G and S3). These findings suggest that GRP78 positively regulates breast CSCs.

**FIGURE 1:**
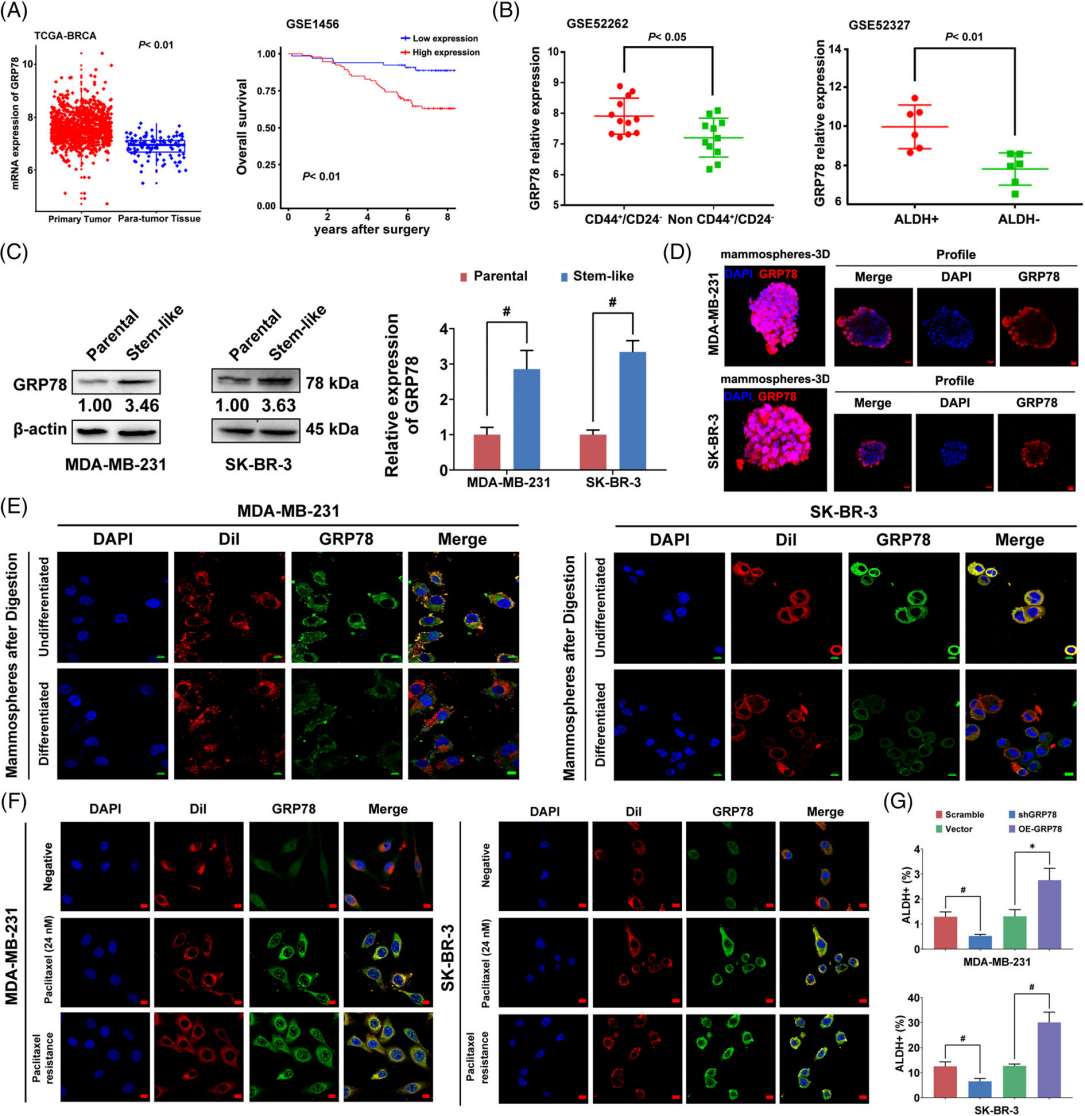
GRP78 positively regulates breast CSCs. (A) Comparative analysis of GRP78 gene expression between the primary tumour tissues and para‐tumour tissues from a cohort of TCGA breast cancer patients (upper panel). Overall survival curves were constructed according to GRP78 levels using the GEO database (lower panel, GSE1456). (B) GRP78 expression was compared between CD44^+^/CD24^−^ breast CSCs (*n* = 12) and non‐CD44^+^/CD24^−^ ones (*n* = 11) using the GEO database (GSE52262, upper panel). The GRP78 expression of ALDH^+^ breast cancer stem‐like cells (*n* = 6) and ALDH^−^ ones (*n* = 6) were also compared in the GEO database (GSE52327, lower panel). (C) ALDH^+^ cells were isolated as breast cancer stem‐like cells to detect the GRP78 expression and compared with the unsorted breast cancer cells. (D) The fluorescence of GRP78 expression on the surface of the mammospheres was displayed in the confocal 3D imaging (upper panel). The scale bars indicate 20 μm. Fluorescence changes of GRP78 expression following mammospheres differentiation were also detected (lower panel). The scale bars indicate 50 μm. (E) The mammospheres were digested into single cells before performing an immunofluorescence analysis. A part of single‐cell suspensions was collected for immunofluorescence analysis directly (undifferentiated). The other part was differentiated in the plain culture well for 48 h before immunofluorescence detection. Thus, the fluorescence intensities of GRP78 were compared before and after differentiation. The cell membrane was visualised with Dil staining (red) and merged with GRP78 (green). The scale bars indicate 10 μm. (F) Representative fluorescence imaging of GRP78 localisation in paclitaxel treatment or paclitaxel‐resistant MDA‐MB‐231 and SK‐BR‐3 cells. The cell membrane was visualised with Dil staining (red) and merged with GRP78 (green). The scale bars indicate 10 μm. (G) ALDH^+^ cells were detected in GRP78 overexpression and knockdown cells compared with their empty vector or scrambled shRNA control. Data were represented as mean ± SD. For statistical analysis, Wilcoxon test (A) and unpaired two‐sided Student's *t* test (B, C, G) were applied. ^*^ *p* < .05, ^#^ *p* < .01. OE‐GRP78: GRP78 overexpression; shGRP78: GRP78 knockdown

Small molecule microarrays are emerging as valuable tools for high‐throughput screening in drug discovery. We printed 1836 kinds of small molecules on a surface plasmon resonance (SPR) slide to identify the potential inhibitor (Figure 2A). Through screening, twelve compounds were shown to have the potential binding interaction. Notably, cefoselis had the strongest binding affinity (Figure 2B). The result was further validated by isothermal titration calorimetry (ITC) technology, indicating that hydrophobic and van der Waals forces jointly contributed to the interaction between cefoselis and GRP78 (Figure 2C and D). In addition, FITC‐labelled cefoselis and Alexa Fluor 555‐coupled GRP78 were colocalised in breast cancer cell lines. Colocalisation of GRP78 and cefoselis was primarily found in the cytoplasm before paclitaxel treatment. However, the unfolded protein response (UPR) is activated following paclitaxel treatment. The UPR sensor GRP78 had been reported to translocate toward the cell membrane to bind with ligands and facilitate cell survival.^9^ Correspondingly, GRP78 presented colocalisation with cefoselis at the cell surface upon paclitaxel treatment (Figure 2E). CETSA analysis suggested that cefoselis improved the thermal stability of GRP78, further validating the binding between them in breast cancer cells (Figure 2F).

**FIGURE 2:**
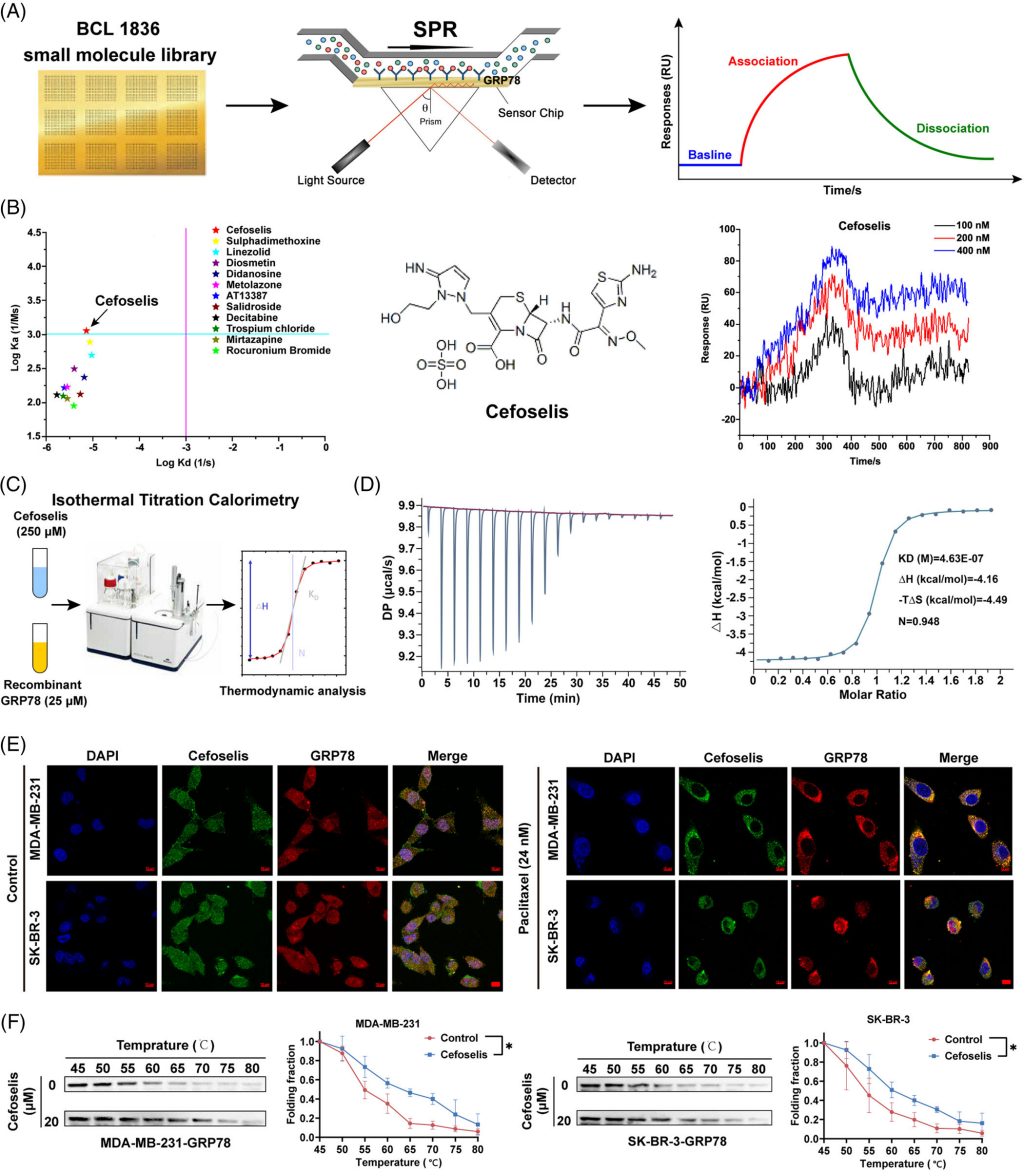
Screening and identification of cefoselis as a GRP78‐targeting agent. (A) BCL (1836) small molecule library was printed on the surface plasmon resonance (SPR) slide to screen the candidate GRP78‐targeting agent. (B) A total of 12 compounds were screened with the potential binding interaction. Cefoselis was identified as the strongest compound binding with GRP78 in a dose‐dependent manner. (C) Isothermal titration calorimetry (ITC) assay was performed to measure the binding affinity of cefoselis with the recombinant GRP78 protein. (D) The raw and integrated heat release in the ITC assay were fitted to obtain the binding parameters. (E) Cefoselis was covalently coupled to FITC (green), and GRP78 was labelled using Alexa Fluor 555 (red). The colocalisation of cefoselis and GRP78 in control or paclitaxel‐induced breast cancer cells was observed by immunofluorescence. (F) Cellular thermal shift assay (CETSA) of the thermal stability of the GRP78 in cell lysate after cefoselis (20 μM) treatment. ANOVA for repeated measurements was applied. ^*^ *p* < .05

As expected, cefoselis efficiently reduced paclitaxel‐induced upregulation of breast cancer stem‐like cells (Figures 3A and S4A‐D). Meanwhile, cefoselis dose‐dependently limit the number and size of mammospheres in breast cancer cell lines (Figures 3B and S4E). Besides, the paclitaxel‐induced overactivation of LRP5/β‐catenin signalling was suppressed by cefoselis (Figures 3C and S4F), which is independent of general protein synthesis inhibition (Figure S4G). Correspondingly, cell viability, colony formation, transwell and wound‐healing assays demonstrated the synergistic effects between cefoselis and paclitaxel (Figure S5). In vivo, cefoselis significantly limited the tumourigenicity of breast CSCs sorted from SK‐BR‐3 cells in NOD/SCID mice in a dose‐dependent manner (Figure 3D). In addition, cefoselis significantly promoted paclitaxel chemosensitivity to limit breast cancer growth (Figure 3E). A lung colonisation model further validated that cefoselis synergistically interacted with paclitaxel to inhibit MDA‐MB‐231 growth in the lung, accompanied by the reduction of metastatic lesions in the combination group (Figures 3F and S6A). The flow cytometry assay revealed that the population of breast cancer stem‐like cells increased by paclitaxel was significantly suppressed by cefoselis (Figures 3G and S6B). Consistent with in vitro findings, the LRP5/β‐catenin signalling was suppressed by cefoselis (Figure 3H), and paclitaxel‐induced apoptosis was aggravated (Figure S6C). These findings highlight cefoselis as a potential CSCs‐limiting agent to improve breast cancer prognosis.

**FIGURE 3:**
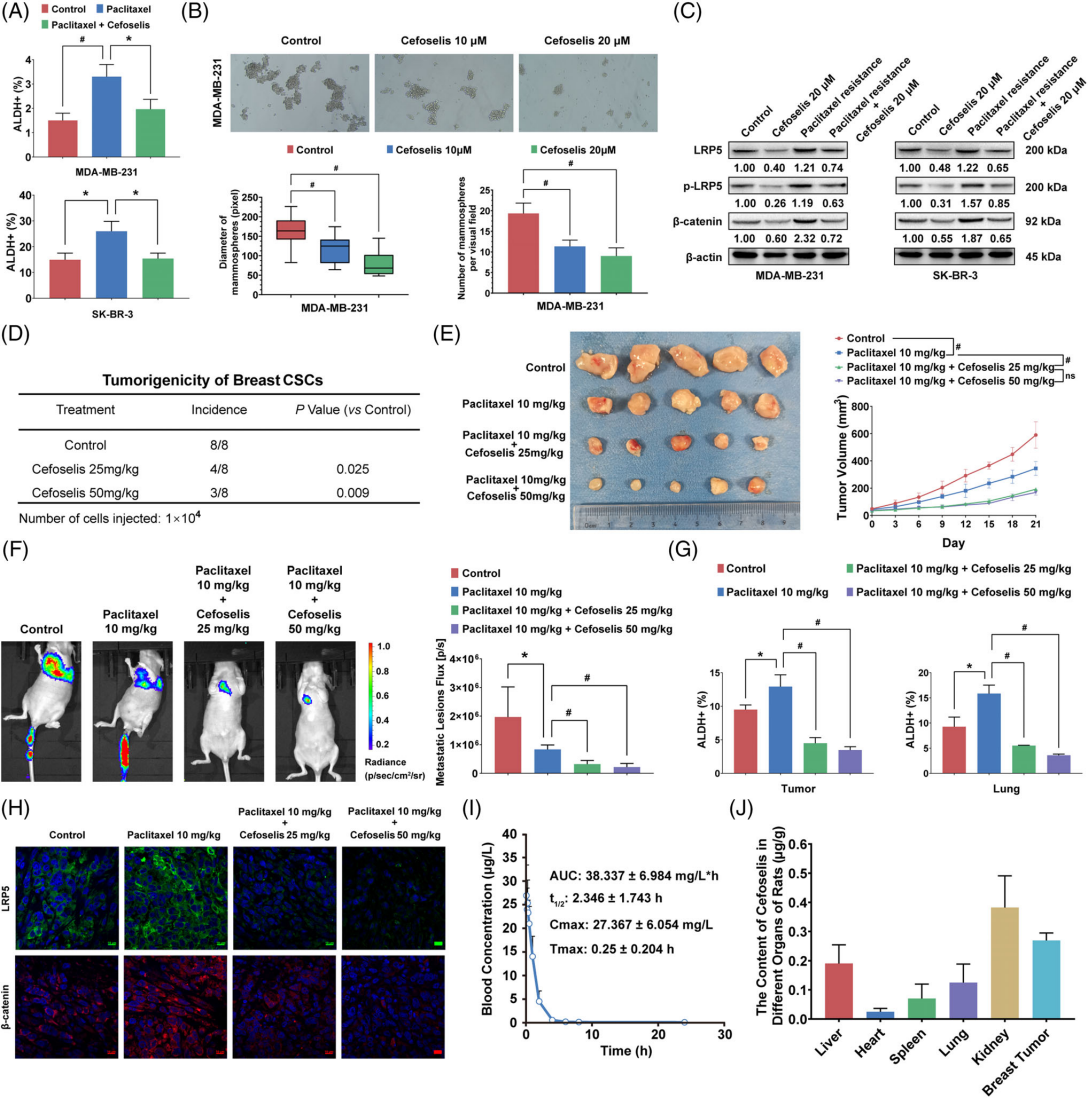
Cefoselis limits breast CSCs with high safety and targeted characteristic. (A) ALDH^+^ frequency of MDA‐MB‐231 and SK‐BR‐3 was detected by flow cytometry following treatment with paclitaxel (24 nM) alone or cefoselis (20 μM) combination for 6 h. (B) MDA‐MB‐231 and SK‐BR‐3 cells were cultured in ultralow attachment plates with the conditioned medium for mammosphere formation. The number and size of mammospheres in both cell lines were quantified under 10 and 20 μM cefoselis treatment. (C) LRP5, p‐LRP5, and β‐catenin expression were measured in paclitaxel‐resistant cells following cefoselis (20 μM) treatment. (D) The CD44^+^/CD24^−/low^ subpopulation of SK‐BR‐3 cells was sorted and inoculated into the mammary fat pads of NOD/SCID mice at the density of 1×10.^4^ The tumour incidence was identified and quantified following treatment with 25 and 50 mg/kg cefoselis (*n* = 8). (E) Orthotopic breast cancer xenograft was established as mentioned above. The left panel is the representative image of tumours separated from Balb/c nude mice. The right panel is the tumour growth curve (*n* = 5). (F) The bioluminescence of the lung colonisation was imaged (left panel) and quantified (right panel) (*n* = 4). (G) ALDH^+^ stem‐like cells in primary tumour tissues and lung metastasis lesions were analysed by flow cytometry (*n* = 3). (H) LRP5 and β‐catenin expression in primary tumour tissues of each group were detected by immunofluorescence assay (the scale bars indicate 10 μm). (I) SD rats (*n* = 6) were injected with 50 mg/kg cefoselis via tail vein. Pharmacokinetic curves were recorded, and the pharmacokinetic parameters were calculated. (J) Breast cancer‐bearing mice (*n* = 6) were administered 25 mg/kg cefoselis by intraperitoneal injection, and the tissue distribution of cefoselis was detected 1 h later. Data were represented as mean ± SD. For statistical analysis, one‐way ANOVA and Bonferroni as post hoc test (A, B), Wilcoxon test (D), ANOVA for repeated measurements (E), and unpaired Student's *t* tests (F, G) were applied. ^*^ *p* < .05, ^#^ *p* < .01

We next validated the CSCs‐limiting effects and safety of cefoselis on immune‐competent mice. Cefoselis brought little hepatotoxicity, nephrotoxicity, and no aggravation of leukopenia when coadministrated with paclitaxel (Table S3).^5^ The pharmacokinetic study demonstrated that the Cmax and AUC_0 –_ *_t_* values of cefoselis were 27.367 mg/L and 38.337 mg/L·h, and the *t*_1/2_ was determined as 2.346 h (Figure 3I). Moreover, cefoselis had a relatively high concentration in breast tumours, just behind the kidney (Figure 3J). In agreement with the previous results, cefoselis synergistically facilitated paclitaxel to inhibit breast cancer growth and lung metastasis, and reduced ALDH1A3 activity induced by paclitaxel, as well as the expression of LRP5 and β‐catenin (Figure S7). These findings demonstrate that cefoselis could be safely used as an adjuvant agent during chemotherapy with a natural tendency to the breast.

Mechanistically, it was found that the inhibition of cefoselis on stem‐like cells and tumourigenicity of breast CSCs were relieved following GRP78 or LRP5 overexpression (Figures 4A and B and S8). Meanwhile, GRP78 overexpression resulted in an enhanced expression of phosphorylated‐LRP5/β‐catenin signalling under the Wnt inhibitor treatment in breast cancer cell lines, suggesting a possible interaction between GRP78 and LRP5 (Figures 4C and S9A).^10^ Coimmunoprecipitation (Co‐IP) assay further indicated an interaction between GRP78 and LRP5, which was enhanced by paclitaxel treatment or chemoresistance (Figure 4D). Co‐IP of different truncations of LRP5 with GRP78 confirmed that the binding site located in LRP5 fragment 201–400 (Figure 4E–F). Molecular docking analysis suggested the highest binding energy was attributed to PHE294 of LRP5 (Figure 4G–H). The binding between GRP78 and LRP5 was abrogated following PHE294 mutation (Figures 4I and S9B). Notably, cefoselis also displayed strong binding with PHE294 (Figures 4J and S9C). Cefoselis interfered with the binding between GRP78 and LRP5 and attenuated their interaction enhanced by paclitaxel (Figure 4K). Moreover, the frequency of breast cancer stem‐like cells declined by cefoselis was abolished due to the mutation of PHE294 (Figure S9D). Therefore, cefoselis limits breast CSCs mainly by interrupting the binding between GRP78 and LRP5.

**FIGURE 4:**
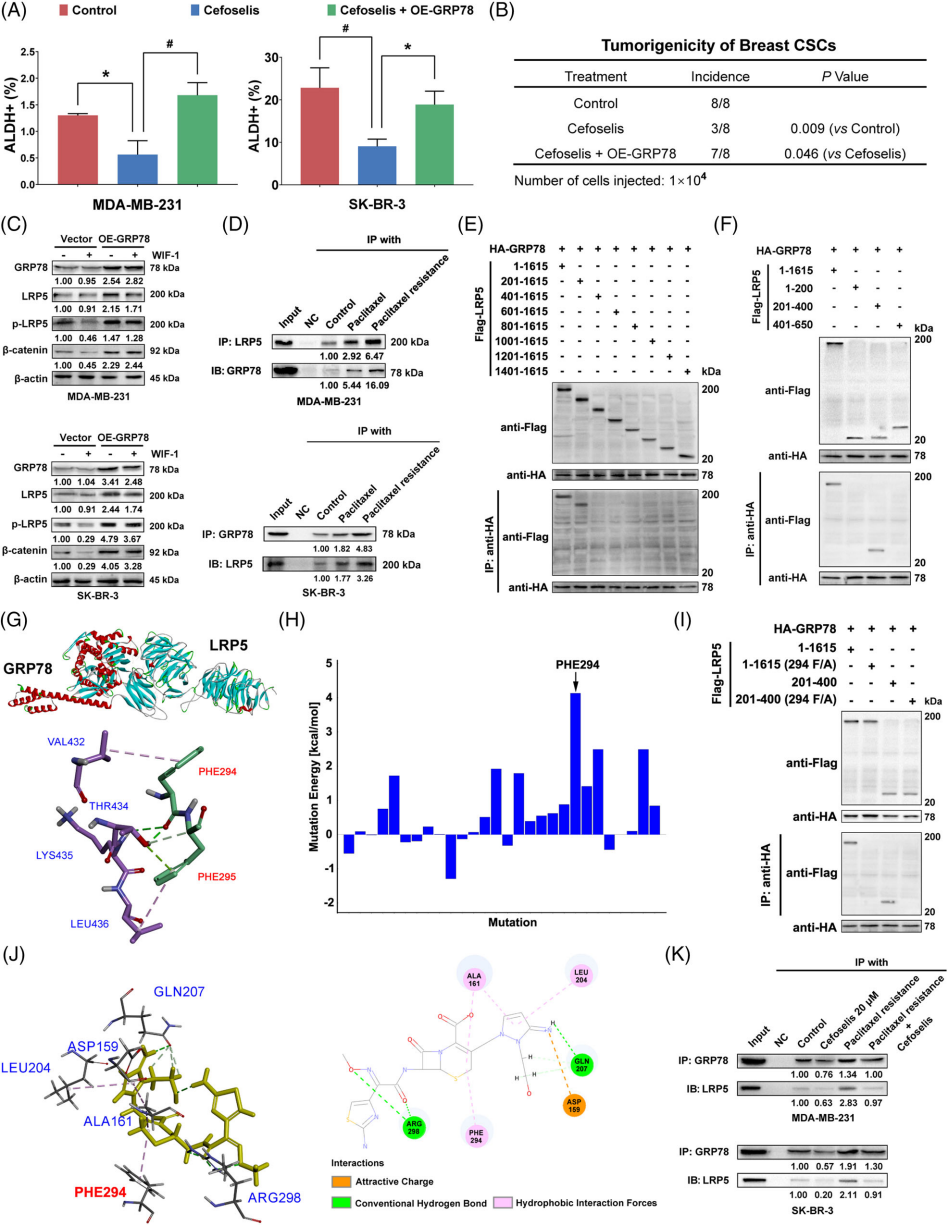
Cefoselis disrupts the interaction between GRP78 and LRP5 via PHE294. (A) ALDH^+^ frequency of breast cancer cells with GRP78 overexpression was detected following cefoselis (20 μM) treatment. (B) The CD44^+^/CD24^−/low^ subpopulation of control or GRP78 overexpressing SK‐BR‐3 cells was sorted and inoculated into the mammary fat pads of NOD/SCID mice at the density of 1×10.^4^ The tumour incidence was identified and compared following cefoselis (25 mg/kg) treatment (*n* = 8). (C) β‐catenin, LRP5 and its phosphorylation levels were measured in MDA‐MB‐231 and SK‐BR‐3 cell lines following GRP78 overexpression under the administration of Wnt inhibitor WIF‐1 (1 μg/ml) for 24 h. (D) The interaction of GRP78 with LRP5 was analysed in paclitaxel‐resistant and paclitaxel‐treated (24 nM) MDA‐MB‐231 and SK‐BR‐3 cells by Co‐IP assays. (E) The full length of LRP5 amino acids was truncated into every 200 fragments and labelled with a Flag to coprecipitate with HA‐labelled GRP78. The successful construction of the recombinant plasmids was verified by western blot using anti‐Flag and anti‐HA antibodies. Co‐IP was then performed using anti‐HA coupled resin. (F) The LRP5 fragments, including 1–200, 201–400, and 401–650, were synthesised to coprecipitate with HA‐labelled GRP78 to confirm the binding sites. (G) The binding mode between GRP78 and LRP5 was performed by molecular docking analysis. (H) The mutation energy was estimated if the binding site was mutated. (I) Co‐IP analysis was performed to verify the interaction between GRP78 and LRP5 when PHE294 of LRP5 was mutated to alanine. (J) The interaction forces, sites, and types between cefoselis and LRP5 were analysed by molecular docking. (K) The interaction of GRP78 with LRP5 was analysed in MDA‐MB‐231 and SK‐BR‐3 cells following cefoselis (20 μM) treatment. Their interactions in the paclitaxel‐resistant cells were also analysed following cefoselis administration. Data were represented as mean ± SD. For statistical analysis, one‐way ANOVA and Bonferroni as post hoc test (A) and Wilcoxon test (B) were applied. ^*^ *p* < .05, ^#^ *p* < .01. OE‐GRP78: GRP78 overexpression

In conclusion, GRP78/LRP5/β‐catenin signalling was identified as a novel pathway promoting breast CSCs. Moreover, cefoselis was identified as a GRP78‐targeting agent to enhance breast cancer chemosensitivity and limit metastasis by inhibiting CSCs in vitro and in vivo. Our findings highlight the significance of ER stress signalling in CSC regulation and provide cefoselis as an adjuvant agent to improve breast cancer prognosis by targeting GRP78.

## CONFLICT OF INTEREST

The authors declare that they have no competing interests.

## FUNDING INFORMATION

This work was supported by the National Natural Science Foundation of China (No. 82004373, 82074165, 81873306, 81973526, 82004132, 82174165); State Key Laboratory of Dampness Syndrome of Chinese Medicine (No. SZ2021ZZ19); Guangdong Science and Technology Department (No. 2016A030306025); Science and Technology Planning Project of Guangdong Province (No. 2021A0505030059, 2017B030314166); Department of Education of Guangdong Province (No. 2018KZDXM022, A1‐2606‐19‐111‐009, 2019KQNCX019); The 2020 Guangdong Provincial Science and Technology Innovation Strategy Special Fund (Guangdong‐Hong Kong‐Macau Joint Lab), (No. 2020B1212030006); Traditional Chinese Medicine Bureau of Guangdong Province (No. 20201132, 20211114, 20212085, 20225011); Guangdong Medical Research Foundation (No. 20201119103046743); Guangzhou Science and Technology Project (No. 202102010316, 202102010241, 201904010407); The Specific Research Fund for TCM Science and Technology of Guangdong provincial Hospital of Chinese Medicine (No. YN2018MJ07, YN2018QJ08); the Foundation for Young Scholars of Guangzhou University of Chinese Medicine (No. QNYC20190101); Research Fund for Bajian Talents of Guangdong Provincial Hospital of Chinese Medicine (No. BJ2022KY18, BJ2022KY12).

## Supporting information

Supporting Information

Supporting Information

Supporting Information

Supporting Information

Supporting Information
