# Supplementary material for: Cefoselis enhances breast cancer chemosensitivity by directly targeting GRP78/LRP5 signalling of cancer stem cells
Source: Clin Transl Med. 2023 Feb 19;13(2):e1119. doi: 10.1002/ctm2.1119 (PMC9939292; doi:10.1002/ctm2.1119)
Supplement: Supplementary file 1 — Supporting Information [file CTM2-13-e1119-s005.docx]

**1. Materials and Methods**

**1.1 Cell culture**

Breast cancer cell lines including MCF-7, MDA-MB-231, T47D, BT474, and non-malignant cells including HUVEC, HSF, and HBL-100 were maintained in Dulbecco's Modified Eagle Medium (DMEM, Gibco, NY, USA). BT-549 and SK-BR-3 were maintained in RPMI 1640 medium (Gibco, NY, USA). All cell line were purchased from KeyGEN BioTECH (Nanjing, China). Paclitaxel-resistant breast cancer cells were induced by long-term culture with gradually increasing concentrations of paclitaxel. All these cells were supplemented with 10% fetal bovine serum (Gibco, NY, USA) and 1% penicillin and streptomycin (Gibco, NY, USA) and incubated at 37°C in a humidified incubator containing 5% CO_2_. MCF-10A cells were cultured in DMEM/F12 medium supplemented with 5% horse serum, 1% penicillin and streptomycin (Gibco, NY, USA), 20 ng/ml recombinant human epidermal growth factor (EGF), 100 ng/ml cholera toxin, 10 μg/ml insulin (Sigma-Aldrich, Shanghai, China) and 0.5 μg/ml hydrocortisone (STEMCELL Technologies, Vancouver, Canada). The identities of all these cell lines have been authenticated by short tandem repeat profiling and tested for mycoplasma contamination.

**1.2 Western blot**

Following SDS-PAGE separation, the proteins were transferred to PVDF membranes (Millipore, Billerica, MA, USA) and probed with the primary antibodies, including E-cadherin antibody (20874-1-AP), N-cadherin antibody (22018-1-AP), vimentin antibody (10366-1-AP), GRP78 antibody (11587-1-AP), β-catenin antibody (51067-2-AP), GSK-3β (22104-1-AP), LRP5 (24899-1-AP), Flag (20543-1-AP), HA (51062-2-AP) (Proteintech, Rosemont, IL, USA); β-actin antibody (4970), GAPDH (5174), Akt (4691), p-Akt (4058) (Cell Signaling Technology, Danvers, MA, USA); p-GSK-3β (AP0261, ABclonal Technology Cambridge, Boston, USA); p-LRP5 (AF4345, Affinity Biosciences, Cincinnati, OH, USA). Finally, the bands were imaged through the ECL. Following secondary antibodies incubation, an advanced ECL luminescence reagent (Tanon Science & Technology, Shanghai, China) was used, and optical density measurement was taken by ImageLab software (BIO-RAD, Hercules, CA).

**1.3 Transfection of plasmids**

GRP78 recombinant plasmid was obtained from Vigene Biosciences (Jinan, China). The shRNA plasmid for *GRP78* was purchased from Gene Pharma Co, LTD (Shanghai, China). As directed by the manufacturer, all plasmids were transfected using lipofectamine 3000 (Invitrogen, Carlsbad, CA, USA).

**1.4 Cell viability**

The cell viability was measured by CCK-8 reagent (Beyotime Biotechnology, Shanghai, China) according to the manufacturer's instructions as described before ^1^.

**1.5 Colony formation assay**

Colony formation assays were conducted by seeding 500 cells per well in a 6-well plate. The cells were treated as instructed and cultured for 2 weeks. Staining was done with coomassie blue after the colonies fixed in 4% paraformaldehyde.

**1.6 Wound-healing and transwell invasion assay**

Wound-healing and transwell invasion assays were performed as described before ^2^.

**1.7 Immunofluorescence**

Immunofluorescence was performed as described in our previous studies ^3^.

**1.8 CSCs population analysis and mammosphere formation assay**

Aldehyde dehydrogenase (ALDH) staining was used to detect breast cancer stem-like cells as described before ^3^. ALDH^+^ cells were sorted by a FACS Aria III flow cytometer (BD Biosciences, San Jose, CA, USA) to perform the immunoblotting assay. CD44^+^/CD24^−^ is considered the biomarker of breast CSCs. CD44^+^/CD24^−/low^ subpopulation was quantified by flow cytometry staining with antibodies against PE-conjugated CD24 (12-0247-42) and FITC-conjugated CD44 (11-0441-81) (Thermo Fisher Scientific, Hudson, USA). Mammosphere formation was assessed by culturing single breast cancer cells in DMEM/F12 supplemented with 1% penicillin-streptomycin (Gibco, NY, USA), 2% B27 supplement (Gibco, NY, USA), 20 ng/ml EGF, 5 μg/ml insulin and 0.4% bovine serum albumin (Sigma-Aldrich, Shanghai, China) on ultralow attachment plates. Mammosphere number and size were quantified microscopically.

**1.9 Co-immunoprecipitation analysis**

The full-length LRP5 and a series of truncated LRP5 were introduced into the pCMV3-SP-N-FLAG plasmid. The *GRP78* was introduced into the pcDNA3.1-HA plasmid. The LRP5 sequence of pCMV3-SP-N-FLAG was mutated using a commercial site-directed mutagenesis kit (Agilent (Stratagene), Beijing, China). The mutation results in amino acid substitution of phenylalanine to alanine at amino acid position 294. The sequence integrity was verified in all plasmid constructs by DNA sequencing. These plasmids with Flag-tagged full-length, truncated LRP5, mutated LRP5, and HA-tagged *GRP78* were transfected into the MDA-MB-231 cells. According to the manufacturer's instructions, an immunoprecipitation assay was carried out using the Pierce Co-Immunoprecipitation Kit (Thermo Fisher Scientific, Hudson, NH, USA). In brief, GRP78 or Flag-tag antibodies were immobilized with resin. The immobilized resin was subsequently incubated with MDA-MB-231 cell lysates to detect LRP5 or HA-tag using immunoblotting.

**1.10 Mice procedures**

Five-week-old female Balb/c nude mice, NOD/SCID mice, and Balb/c mice were obtained from the Beijing Vital River Laboratory Animal Technology Co., Ltd. All experimental treatments of mice were reviewed and approved by the supervision of the Institutional Animal Care and Use Committee of Guangdong Provincial Hospital of Chinese Medicine (ethics approval number: 2017027, 2021075). For Balb/c nude mice, each mouse was injected with 5×10^6^ MDA-MB-231 cells into the mammary fat pad to build breast cancer xenografts. When the tumor volume reached approximately 100 mm^3^, mice were randomly divided into different treatment groups (n=5). Cefoselis (25 mg/kg) and paclitaxel (10 mg/kg) were given every three days by intraperitoneal administration. The volume of the tumor was calculated every two days using the formula ([width]^2^ × [length]/2). Luciferase-tagged MDA-MB-231 cells were injected into the tail vein of mice at a density of 2 × 10^5^ to establish the lung colonization model of breast cancer. Starting from the third week, the mice were randomly segregated into control and various treatment groups (n=6). Cefoselis and paclitaxel were administered as mentioned above. For luminescent imaging, mice were anesthetized with isoflurane and injected with 150 mg/kg D-luciferin (PerkinElmer, Boston, USA). IVIS-spectrum system (PerkinElmer, Boston, USA) was used to image and quantify the bioluminescence of the lung colonization. For tumorigenesis assay, the CSCs were sorted from SK-BR-3 cells by MACS separators according to the manufacturer's protocol of CD44 MicroBeads (130-095-194, Miltenyi Biotec, Guangzhou, China) and CD24 MicroBead Kit (130-095-951, Miltenyi Biotec, Guangzhou, China). The CD44^+^/CD24^−/low^ subpopulation was quantified, collected, and defined as human breast CSCs to subject to a tumorigenesis assay as we previously reported ^4^. Sorted human breast CSCs were resuspended in matrigel (BD Biosciences, San Jose, CA, USA) and inoculated into the mammary fat pads of NOD/SCID mice. In the end, the tumor incidence was identified and counted. In addition, an orthotopic murine breast-cancer model was established by injecting luciferase-tagged 4T1 cells into the mammary fat pads of Balb/c mice. The safety and efficacy of cefoselis were verified in this model by evaluating the biochemical blood indexes, liver, and kidney function, as well as the tumor size and fluorescence intensity in metastatic tissue. For the tissue distribution study of cefoselis, liver, heart, spleen, lung, kidney, and breast tumors of mice were collected following intraperitoneal administration of cefoselis for 1 h. The tissue samples were ground with saline and then treated to remove protein. The supernatant was taken for LC-MS analysis.

**1.11 Pharmacokinetic analysis**

SD rats were obtained from the Beijing Vital River Laboratory Animal Technology Co., Ltd. Experimental treatments of all rats were reviewed and approved by the Institutional Animal Care and Use Committee of Guangdong Provincial Hospital of Chinese Medicine (ethics approval number:2021076). Prior to cefoselis (50 mg/kg) tail vein injection, all rats (n=6) fasted for 12 h with access to water freely. After administration, blood samples were collected at 0, 0.167, 0.333, 0.083, 2, 4, 6, 8, 24 h. The plasma obtained was deproteinized and centrifuged. The supernatant was analyzed by LC-MC. Pharmacokinetic parameters of cefoselis were calculated with the DAS (Drug and Statistics) 2.0 pharmacokinetic software.

**1.12 Immunohistochemistry, hematoxylin-eosin staining and TUNEL analysis**

The tissue microarray (HBre-Duc140Sur-01) was purchased from Shanghai Outdo Biotech Co., LTD. (Shanghai, China), including 118 breast cancer cases. The immunohistochemical analysis followed the protocol described previously ^5^. Hematoxylin and eosin staining was carried out using the Hematoxylin and Eosin Staining Kit (Beyotime Biotechnology, Shanghai, China). The presence of fragmented DNA was detected by TUNEL analysis according to the manufacturer's instruction ^6^. Apoptosis *in situ* was measured by fluorescence intensity.

**1.13 Protein-protein docking analysis**

To process *in silico* interaction between GRP78 and LRP5, Biovia Discovery Studio 2016 software (BIOVIA, San Diego, CA, USA) was applied for the simulation. The 3D structure of GRP78 was acquired from PDB with ID 5E84 ^7^. The structure of LRP5 was constructed in Discovery Studio 2016 through homology modeling based on the structure of PDB with ID 4DG6 ^8^. Following dock protein (ZDOCK) and a further refined protein (RDOCK) procedure, a virtual alanine scan protocol was performed to calculate the mutation energy of amino acid in the binding site between GRP78 and LRP5. In the flexible docking procedure, amino acids which were critical for GRP78-LRP5 interaction were set as the binding site to analyze the interaction between cefoselis and the GRP78-LRP5 complex.

**1.14** **Surface plasmon resonance imaging**

Three-dimensional (3D) photo-cross-linker sensor chip printed with BCL (1836) small molecule library was designed by Guangzhou Gaotong Biological Technology Co., Ltd. The chip was dried under vacuum, and then the photo-crosslinking reactions were performed, followed by shaking and washing with *N, N*-Dimethylformamide (DMF), C_2_H_5_OH, and H_2_O for 15 min, and dried under nitrogen. Purified recombinant protein GRP78 was prepared by WZ Biosceences Inc. and dissolved in PBS pH7.4 containing 20 mM Tris, 50 mM KCl, 5 mM MgCl2, 1 mM DTT. Different concentrations of GRP78 protein (100, 200, and 400 nM) were used as an analyte to get accurate kinetic parameters. The screening was analyzed by a PlexArray® HT SPR system (Plexera Inc., Seattle, DC, USA). Glycine-HCl pH2.0 solution was used to regenerate the surface and remove bound proteins from the sensor chip enabling the slide for additional analyte injections.

**1.15** **Isothermal titration calorimetry assay**

The purified recombinant GRP78 protein was firstly subjected to microdialysis to eliminate the interference of the buffer solution and retain the dialysis tail fluid. Cefoselis (250 μM) was prepared using the dialysis tail fluid, and recombinant GRP78 protein solution was diluted to 25 μM. Before titration, the MicroCal^TM^ PEAQ Isothermal Titration Calorimetry (Malvern Instruments Limited, Shanghai, China) was cleaned according to the procedure, and the instrument state was checked by water titration. When the baseline of water titration was smooth, the area of each peak was relatively close, and the peak height did not exceed 0.05, the titration of samples was performed. 300 μl GRP78 protein solution was absorbed by the syringe slowly, and the bubbles were ensured to eliminate before adding to the sample cell. About 75 μl of cefoselis was added into the sample tube, and the titration would be started automatically.

**1.16 Cellular thermal shift assay (CETSA)**

The CETSA assay was performed as described ^9^. In brief, the cefoselis-treated or control cells were harvested and supplemented with the complete protease inhibitor cocktail. The cell suspensions were freeze-thawed three times using liquid nitrogen. The obtained lysates of cefoselis-treated cells were incubated with cefoselis for 30 min at room temperature. Cefoselis-treated and control cell lysates were divided into smaller (50 µl) aliquots and heated individually at different temperatures (range of 45-80 ℃) for 3 min using a thermal cycler (BIO-RAD, Hercules, CA), followed by cooling for 3 min at room temperature. The heated lysates were centrifuged at 20000 g for 20 min at 4°C in order to separate the soluble fractions from precipitates. The supernatants were analyzed by western blot as mentioned previously.

**1.17 Bioinformatics analysis**

1215 cases with RNA-seq data were downloaded from the TCGA database. Clinical and molecular information was correspondingly obtained. mRNA expression of 6 BRCA datasets was obtained from the Gene Expression Omnibus (GEO) repository. The survival distributions were described by the Kaplan-Meier survival curve, and the log-rank test was used to test the statistical significance. The overall survival and recurrence free survival curves were introduced using the survival package. The correlations between the genes were calculated by Pearson correlation analysis. The Student t-test and one-way ANOVA were used to assess differences in variables between groups. Patients with missing information were excluded from the corresponding analysis. All statistical analyses were performed in the R programming environment (version 3.5.2) and Bioconductor. A *p*-value < 0.05 is considered as significant. All statistical tests were two-sided.

**1.18 Statistical analysis**

Data were presented as mean ± standard deviation (SD). Student's t-test analysis, one-way ANOVA, and the Bonferroni post hoc test were applied. ANOVA for repeated measurement was performed towards repeated measures data. The nonparametric test was used if data were not normally distributed. Survival was analyzed using χ2 analysis on the Kaplan-Meier survival curves. *P* < 0.05 was considered statistically significant.

**References**

1. Zheng YF, Dai Y, Liu WP, et al. Astragaloside IV enhances taxol chemosensitivity of breast cancer via caveolin-1-targeting oxidant damage. *Journal of Cellular Physiology*. Apr 2019;234(4):4277-4290.

2. Zheng YF, Liu PX, Wang N, et al. Betulinic Acid Suppresses Breast Cancer Metastasis by Targeting GRP78-Mediated Glycolysis and ER Stress Apoptotic Pathway. *Oxidative Medicine and Cellular Longevity*. Aug 2019;2019:15. 8781690.

3. Zheng Y, Zhang J, Huang W, et al. Sini San Inhibits Chronic Psychological Stress-Induced Breast Cancer Stemness by Suppressing Cortisol-Mediated GRP78 Activation. *Frontiers in Pharmacology*. 2021-November-29 2021;12(3345)

4. Wang N, Wang Z, Peng C, et al. Dietary compound isoliquiritigenin targets GRP78 to chemosensitize breast cancer stem cells via beta-catenin/ABCG2 signaling. *Carcinogenesis*. Nov 2014;35(11):2544-54.

5. Zheng Y, Liu P, Wang N, et al. Betulinic Acid Suppresses Breast Cancer Metastasis by Targeting GRP78-Mediated Glycolysis and ER Stress Apoptotic Pathway. *Oxid Med Cell Longev*. 2019;2019:8781690.

6. Jiao L, Wang S, Zheng Y, et al. Betulinic acid suppresses breast cancer aerobic glycolysis via caveolin-1/NF-kappaB/c-Myc pathway. *Biochem Pharmacol*. Mar 2019;161:149-162.

7. Yang J, Nune M, Zong Y, Zhou L, Liu Q. Close and Allosteric Opening of the Polypeptide-Binding Site in a Human Hsp70 Chaperone BiP. *Structure*. Dec 1 2015;23(12):2191-2203.

8. Holdsworth G, Slocombe P, Doyle C, et al. Characterization of the interaction of sclerostin with the low density lipoprotein receptor-related protein (LRP) family of Wnt co-receptors. *J Biol Chem*. Aug 3 2012;287(32):26464-77.

9. Martinez Molina D, Jafari R, Ignatushchenko M, et al. Monitoring drug target engagement in cells and tissues using the cellular thermal shift assay. *Science*. Jul 5 2013;341(6141):84-7.
