# Supplementary material for: Cefoselis enhances breast cancer chemosensitivity by directly targeting GRP78/LRP5 signalling of cancer stem cells
Source: Clin Transl Med. 2023 Feb 19;13(2):e1119. doi: 10.1002/ctm2.1119 (PMC9939292; doi:10.1002/ctm2.1119)
Supplement: Supplementary file 2 — Supporting Information [file CTM2-13-e1119-s004.docx]

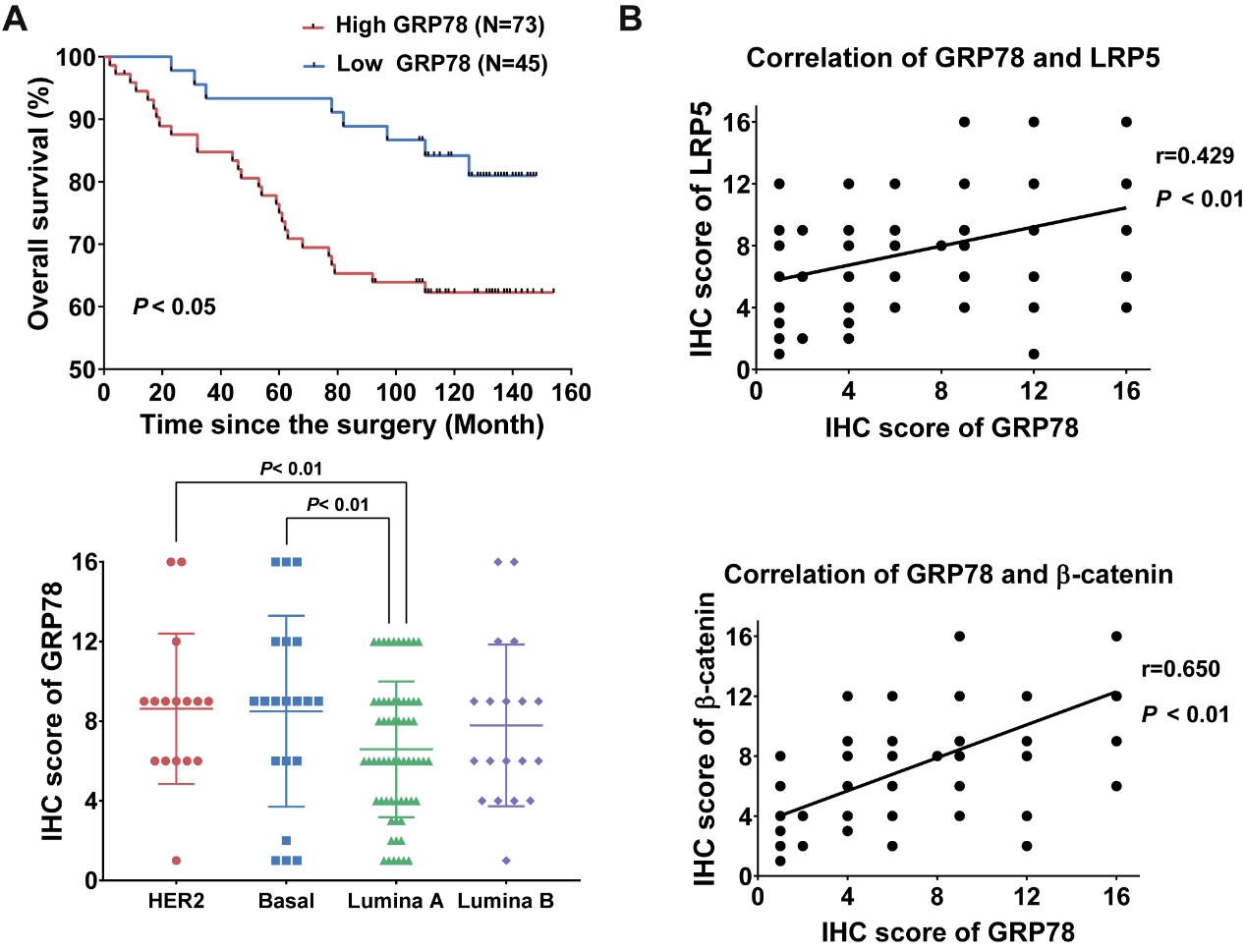
**Figure S1 Tissue microarray analysis of GRP78 in breast cancer. (A)** Upper panel: Kaplan-Meier survival curves were constructed according to GRP78 levels in breast cancer TMA (n=118). Lower panel: GRP78 levels of each breast cancer pathological subtype in the TMA (n=118) were analyzed. **(B)** Linear correlation was examined in breast cancer TMA. Upper panel: Correlation analysis between GRP78 and LRP5 (n=115); Lower panel: Correlation analysis between GRP78 and β-catenin (n=118). Data were represented as Mean ± SD. For statistical analysis, the Wilcoxon test (upper panel of A), Kruskal-Wallis test (lower panel of A), and Spearman correlation analysis (B) were applied.


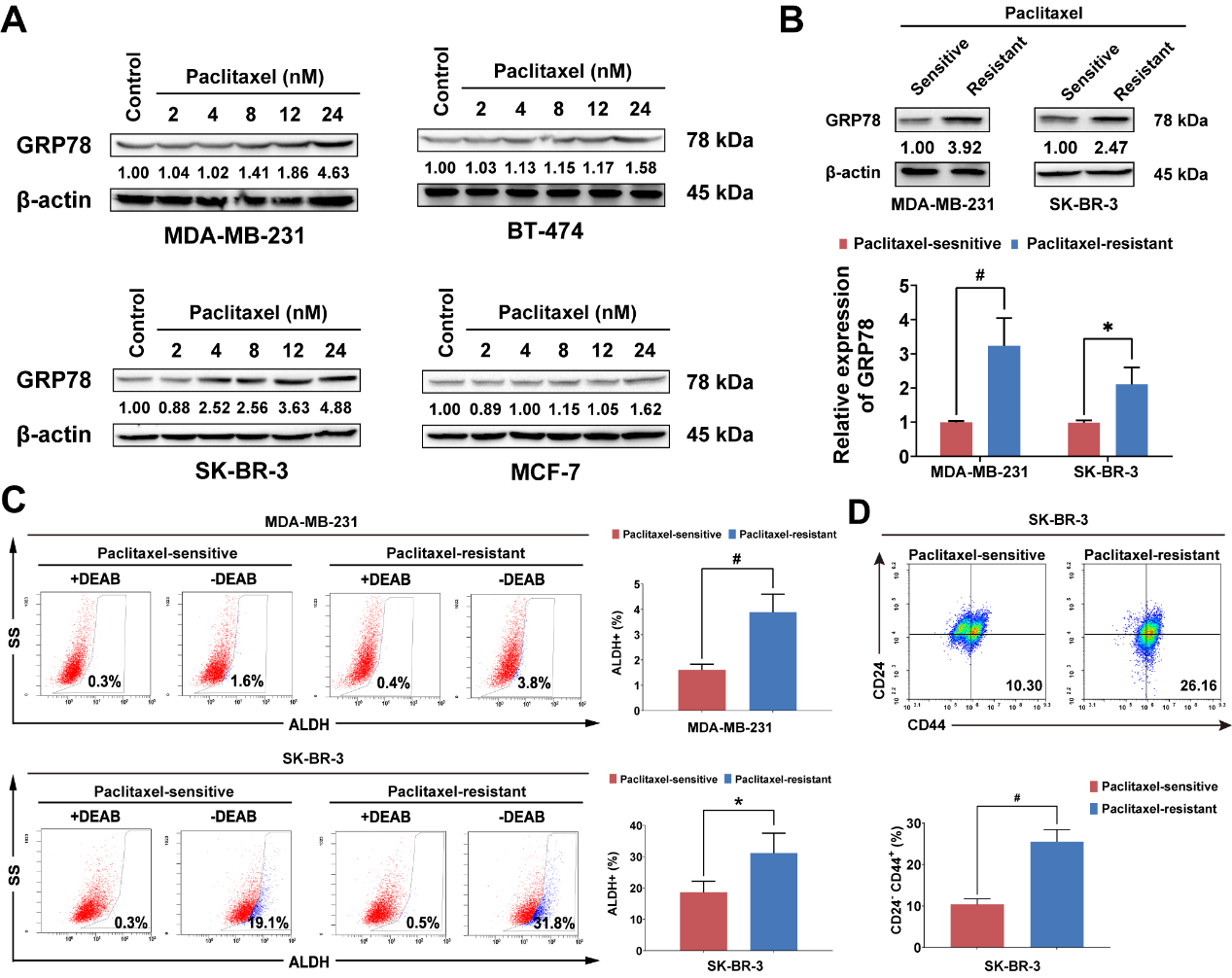
**Figure S2 GRP78 is elevated under paclitaxel treatment and associated with breast CSCs. (A)** Four breast cancer cell lines were treated with a gradient concentration of paclitaxel for 6 h. The corresponding changes of GRP78 expression were detected by Western blot. **(B)** Relative GRP78 expression of paclitaxel-resistant MDA-MB-231 and SK-BR-3 versus paclitaxel-sensitive cells was compared by western blot. **(C)** ALDH^+^ cells in paclitaxel-resistant MDA-MB-231 and SK-BR-3 cell lines were analyzed by flow cytometry. DEAB, a specific inhibitor of ALDH activity, was used to control the background fluorescence. **(D)** The frequency of CD44^+^/CD24^−^ breast CSCs was analyzed in the paclitaxel-resistant SK-BR-3 cell line. Data were represented as Mean ± SD. For statistical analysis, an unpaired Student's t-test was applied. ^*^*P*< 0.05, ^#^*P*< 0.01.


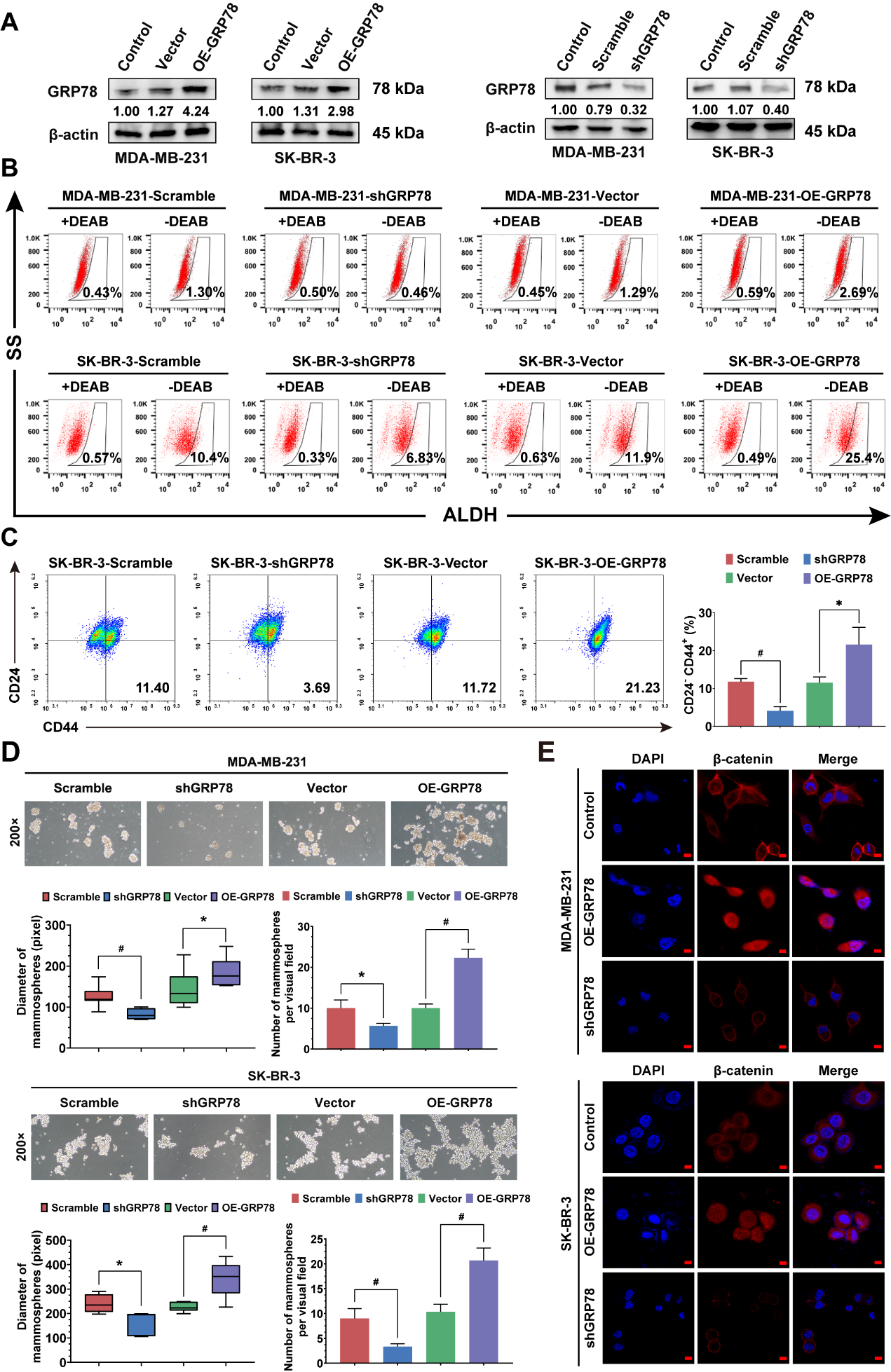
**Figure S3 GRP78 positively regulates breast CSCs. (A)** GRP78 overexpression by gene recombination or knockdown by shRNA in MDA-MB-231 and SK-BR-3 was verified by western blot. **(B)** ALDH^+^ cells were detected in GRP78 overexpression and knockdown cells compared with their empty vector or scrambled shRNA control. Representative flow cytometric diagrams were shown. **(C)** CD44^+^/CD24^−^ breast CSCs population in SK-BR-3 cell line was quantified following knockdown or overexpression of GRP78. **(D)** MDA-MB-231 and SK-BR-3 cells were cultured in ultralow attachment plates with the conditioned medium for mammosphere formation. The number and size of mammospheres influenced by GRP78 overexpression or knockdown in both cell lines were quantified. **(E)** Representative fluorescence image of β-catenin nuclear localization following GRP78 overexpression or knockdown (the scale bars indicate 10 μm). The cell nucleus was counterstained with DAPI (blue), and β-catenin was labeled by red fluorescence. The nuclear localization was indicated by the overlap of fluorophores (pink). Data were represented as Mean ± SD. For statistical analysis, unpaired Student's t-tests were applied. ^*^*P*< 0.05, ^#^*P*< 0.01. OE-GRP78: GRP78 overexpression; shGRP78: GRP78 knockdown.


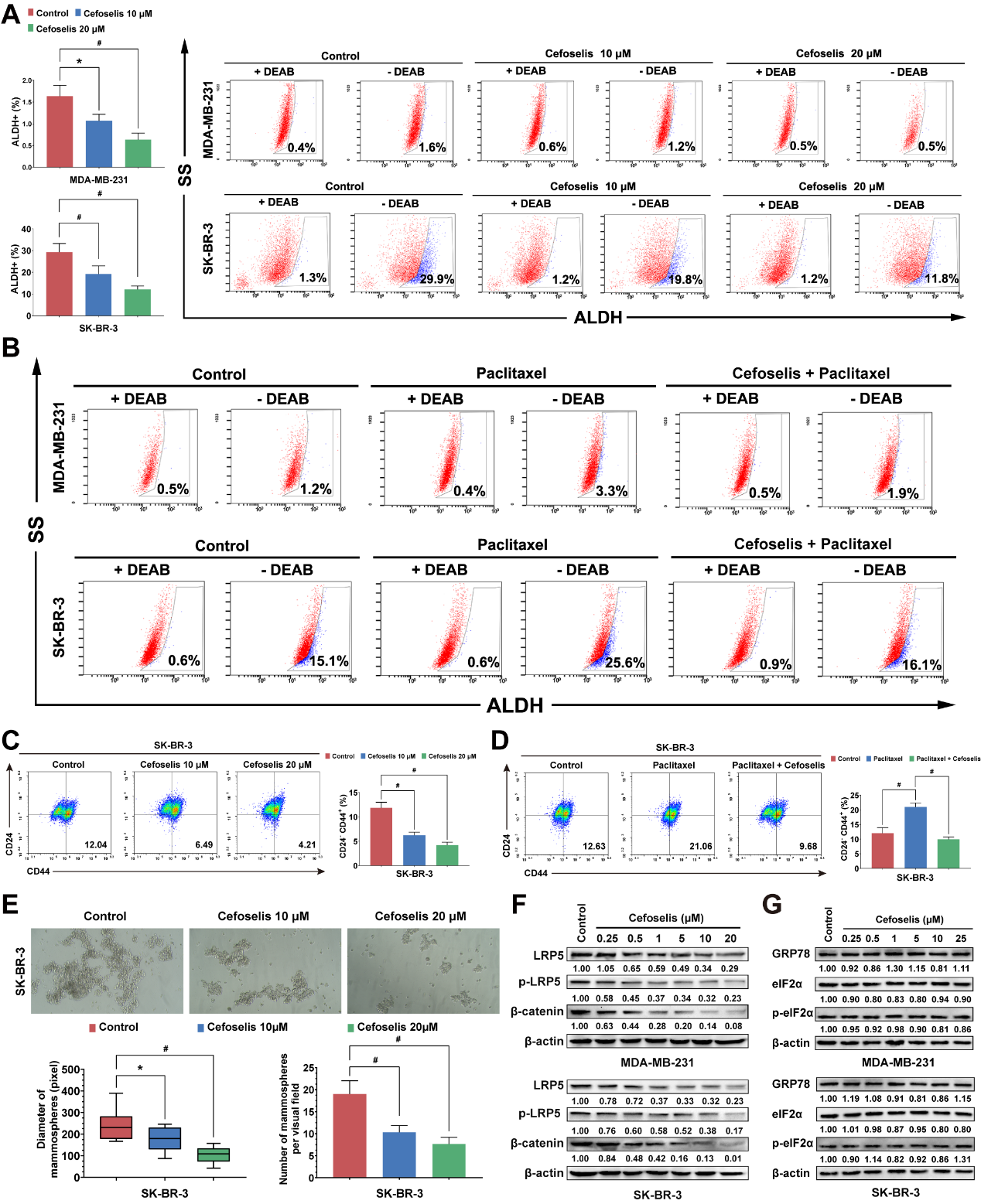
**Figure S4 Cefoselis inhibits breast cancer stemness *in vitro*. (A)** ALDH^+^ cells in MDA-MB-231 and SK-BR-3 cell lines were analyzed following 10 and 20 μM cefoselis treatment for 24 h. **(B)** ALDH^+^ population was detected following treatment with paclitaxel (24 nM) plus with or without cefoselis (20 μM). Representative flow cytometric diagrams were shown. **(C)** The frequency of CD44^+^/CD24^−^ breast CSCs were analyzed in SK-BR-3 cell line treated with 10 and 20 μM cefoselis for 24 h. **(D)** CD44^+^/CD24^−^ breast CSCs population in SK-BR-3 cell line was quantified following treatment with paclitaxel (24 nM) plus with or without cefoselis (20 μM). **(E)** SK-BR-3 cells were cultured in ultralow attachment plates with the conditioned medium for mammosphere formation. The number and size of mammospheres were quantified under 10 and 20 μM cefoselis treatment. **(F)** LRP5, p-LRP5, and β-catenin expression were measured following cefoselis treatment with a gradient concentration. **(G)** GRP78, eIF2α, and p-eIF2α expression were measured following cefoselis treatment with a gradient concentration. Data were represented as Mean ± SD. For statistical analysis, one-way ANOVA and Bonferroni as post hoc test were applied. ^*^*P*< 0.05, ^#^*P*< 0.01.


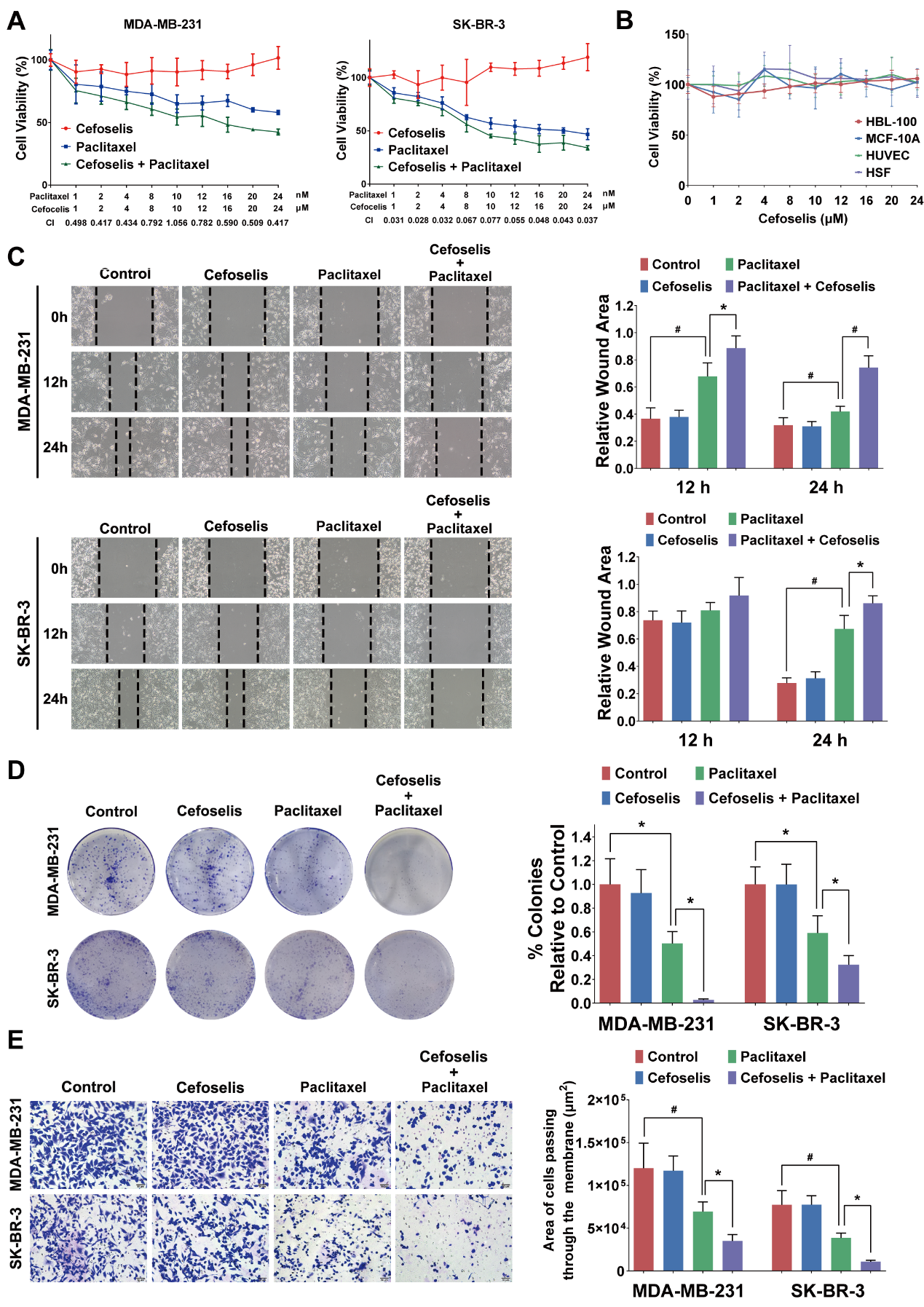
**Figure S5 Cefoselis synergistically enhances paclitaxel chemosensitivity *in vitro*. (A)** MDA-MB-231 and SK-BR-3 cell lines were treated with paclitaxel and cefoselis alone or combination for 48 h. Cell viability was determined by CCK-8 assays. The combination index (CI) for each data point was calculated by the CalcuSyn software (CI values of 0.9-1.1 as additive, 0.3–0.9 as synergistic, and <0.3 as strongly synergistic). **(B)** The cell viability of non-malignant cell lines including HBL-100, MCF-10A, HUVEC, and HSF were measured following cefoselis treatment with a gradient concentration. **(C)** MDA-MB-231 and SK-BR-3 cell lines were scratched and cultured in a serum-free medium following treatment with paclitaxel (4 nM) and cefoselis (20 μM) alone or combination. The wound area was detected at 0, 12, 24 h, respectively. **(D)** MDA-MB-231 and SK-BR-3 cell lines were pre-treated with paclitaxel (4 nM) plus with or without cefoselis (20 μM) for 12 h. A colony formation assay was then performed. **(E)** MDA-MB-231 and SK-BR-3 cells were incubated for 24 h in the matrigel pre-treated chamber following cefoselis (20 μM) with or without paclitaxel (4 nM) treatment. The area of cells that penetrated the filter was analyzed by Image J software. Data were represented as Mean ± SD. For statistical analysis, one-way ANOVA and Bonferroni as post hoc test were applied. ^*^*P*< 0.05, ^#^*P*< 0.01.


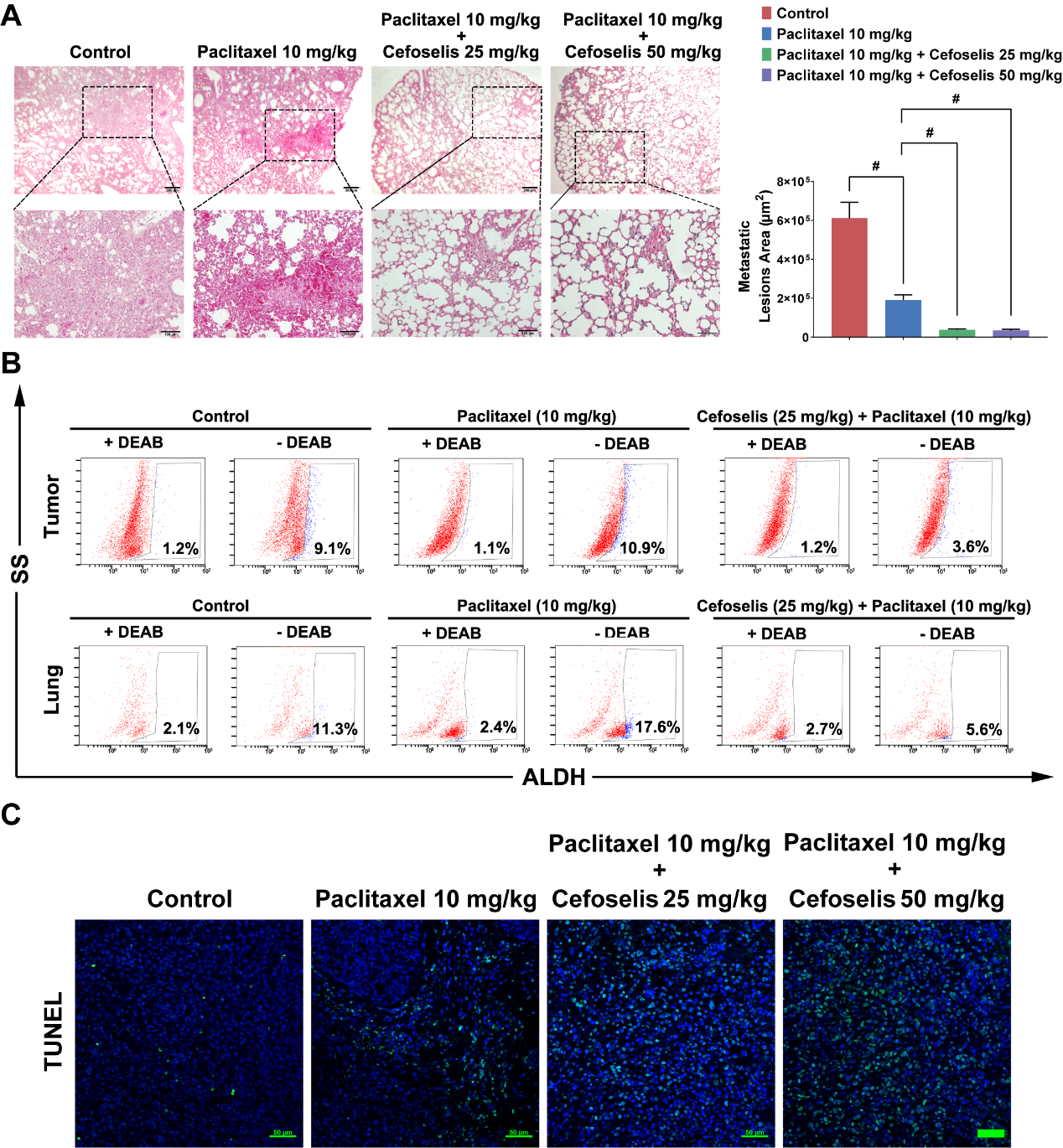
**Figure S6 Cefoselis enhances the anti-breast cancer effects of paclitaxel. (A)** Metastatic lesions of mouse lung tissue were detected by H&E staining. Representative images were shown (left panel), and the area of metastatic lesions was quantified (right panel) (n=4). **(B)** ALDH^+^ stem-like populations in primary tumor tissues and lung metastasis lesions were analyzed in the groups of paclitaxel alone or the combined treatment. Representative flow cytometric diagrams were shown. **(C)** *In situ* analysis of apoptosis by TUNEL staining in breast tumor tissues. Apoptotic cells were marked by green fluorescence (the scale bars indicate 50 μm). Data were represented as Mean ± SD. For statistical analysis, unpaired Student's t-tests (A) were applied. ^*^*P*< 0.05, ^#^*P*< 0.01.


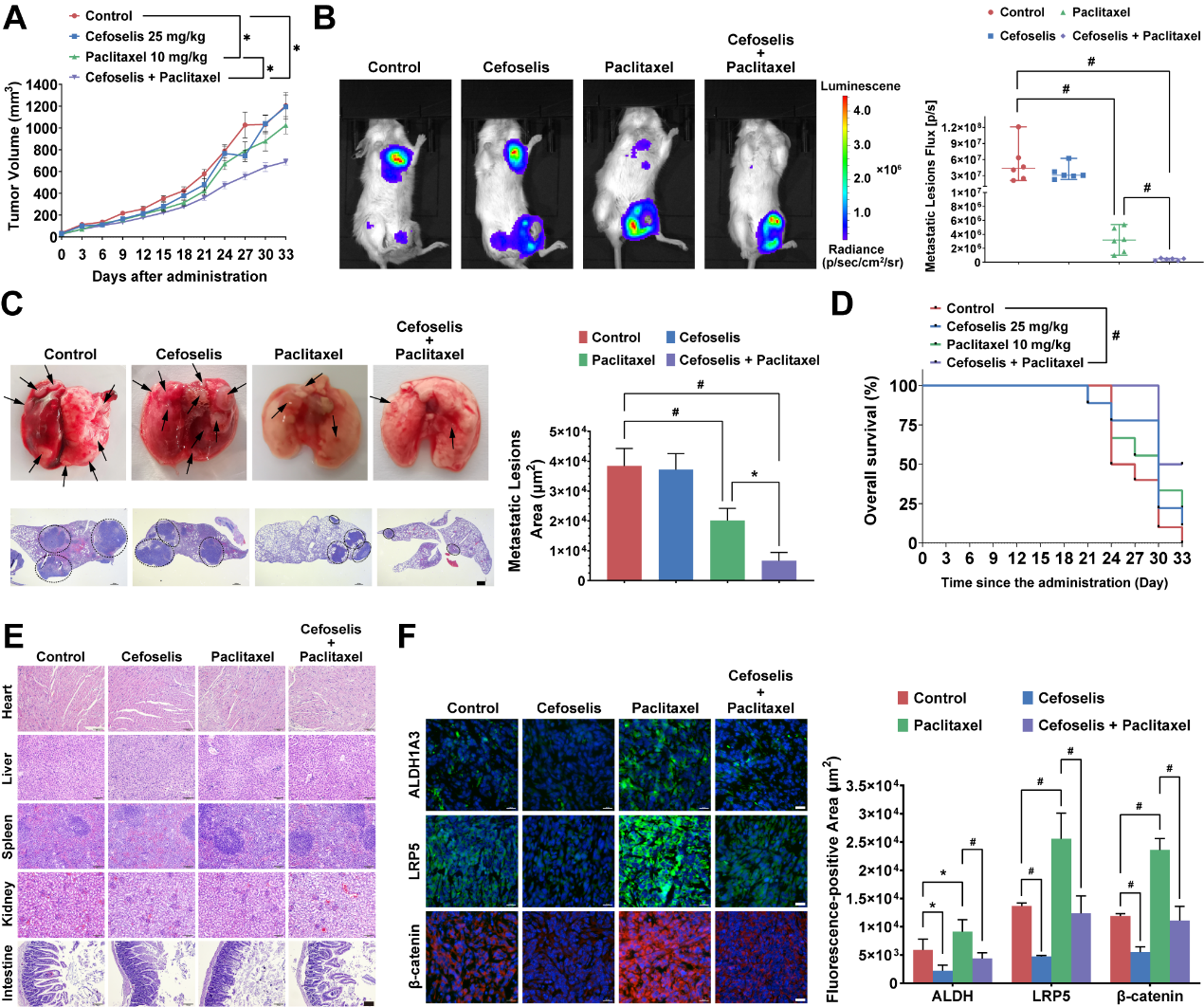
**Figure S7 Therapeutic efficacy and safety of cefoselis in immune competent breast cancer xenografts. (A)** The orthotopic breast cancer BALB/c mouse xenograft was established with 4T1 cell line. The tumor growth curve for each group was plotted (n=10). Cefoselis (25 mg/kg) and paclitaxel (10 mg/kg) were given every three days by intraperitoneal administration. **(B)** *In vivo* imaging of lung metastatic lesions in the breast cancer-bearing mice for each group (left panel). The intensity of bioluminescence was quantified (n=6, right panel). **(C)** Representative pictures of gross lung metastatic lesions in the 4T1 tumor-bearing mice were shown. The black arrow indicates the metastatic lesions. Lung metastatic lesions were also shown by HE staining, and the black circle indicates the metastatic lesions. The scale bars indicate 1000 μm. The metastatic lesion area was quantified by ImageJ software (n=3). **(D)** The survival curves of mice were plotted (n=10). The tumor diameter of 1.3 cm was set as the humane endpoint. **(E)** Representative histopathological pictures of the heart, liver, spleen, kidney, and small intestine of mice in each treatment group. **(F)** The expression of ALDH1A3, LRP5 and β-catenin was detected by immunofluorescence in the tumor tissues of mice (n=4). Data were represented as Mean ± SD. For statistical analysis, ANOVA for repeated measurements (A), Mann-Whitney test (B), Bonferroni as post hoc test (C), Wilcoxon test (D) and unpaired Student's t-tests (F) were applied. ^*^*P*< 0.05, ^#^*P*< 0.01.


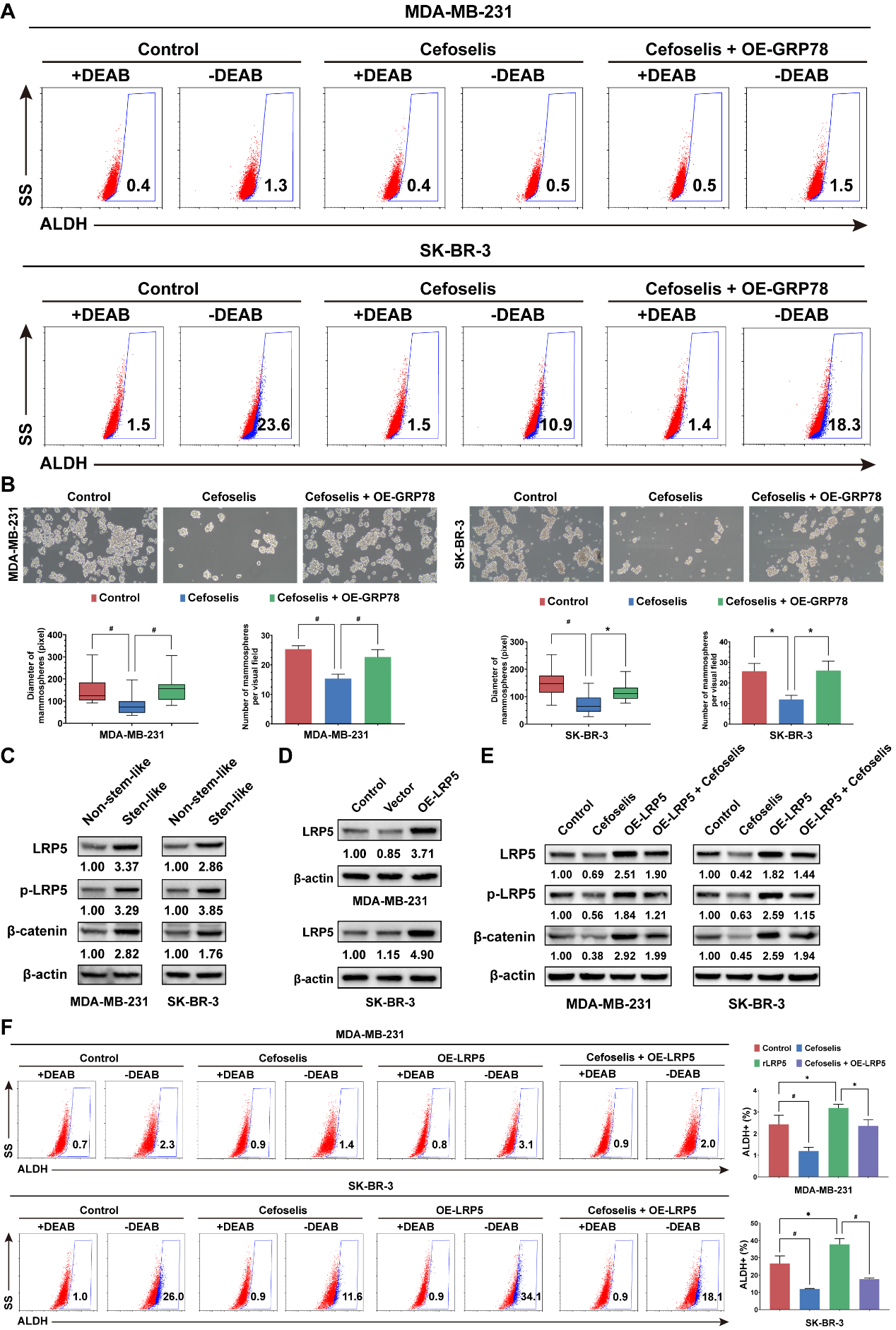
**Figure S8 The inhibition effects of cefoselis on breast cancer cell stemness were relieved following GRP78 or LRP5 overexpression. (A)** ALDH^+^ frequency of breast cancer cells with GRP78 overexpression was detected following cefoselis (20 μM) treatment. Representative flow cytometric diagrams were shown. **(B)** The number and size of mammospheres in breast cancer cells with GRP78 overexpression were quantified under cefoselis (20 μM) treatment. **(C)** ALDH^+^ cells were isolated as breast cancer stem-like cells to detect the LRP5, p-LRP5, and β-catenin expression and compared with the non-stem-like breast cancer cells. **(D)** LRP5 overexpression in MDA-MB-231 and SK-BR-3 was verified by western blot. **(E)** β-catenin expression in LRP5 overexpressing breast cancer cells were measured following cefoselis (20 μM) treatment. **(F)** ALDH^+^ population of breast cancer cells with LRP5 overexpression was detected following cefoselis (20 μM) treatment. Data were represented as Mean ± SD. For statistical analysis, unpaired Student's t-tests were applied. ^*^*P*< 0.05, ^#^*P*< 0.01. OE-GRP78: GRP78 overexpression; OE-LRP5: LRP5 overexpression.


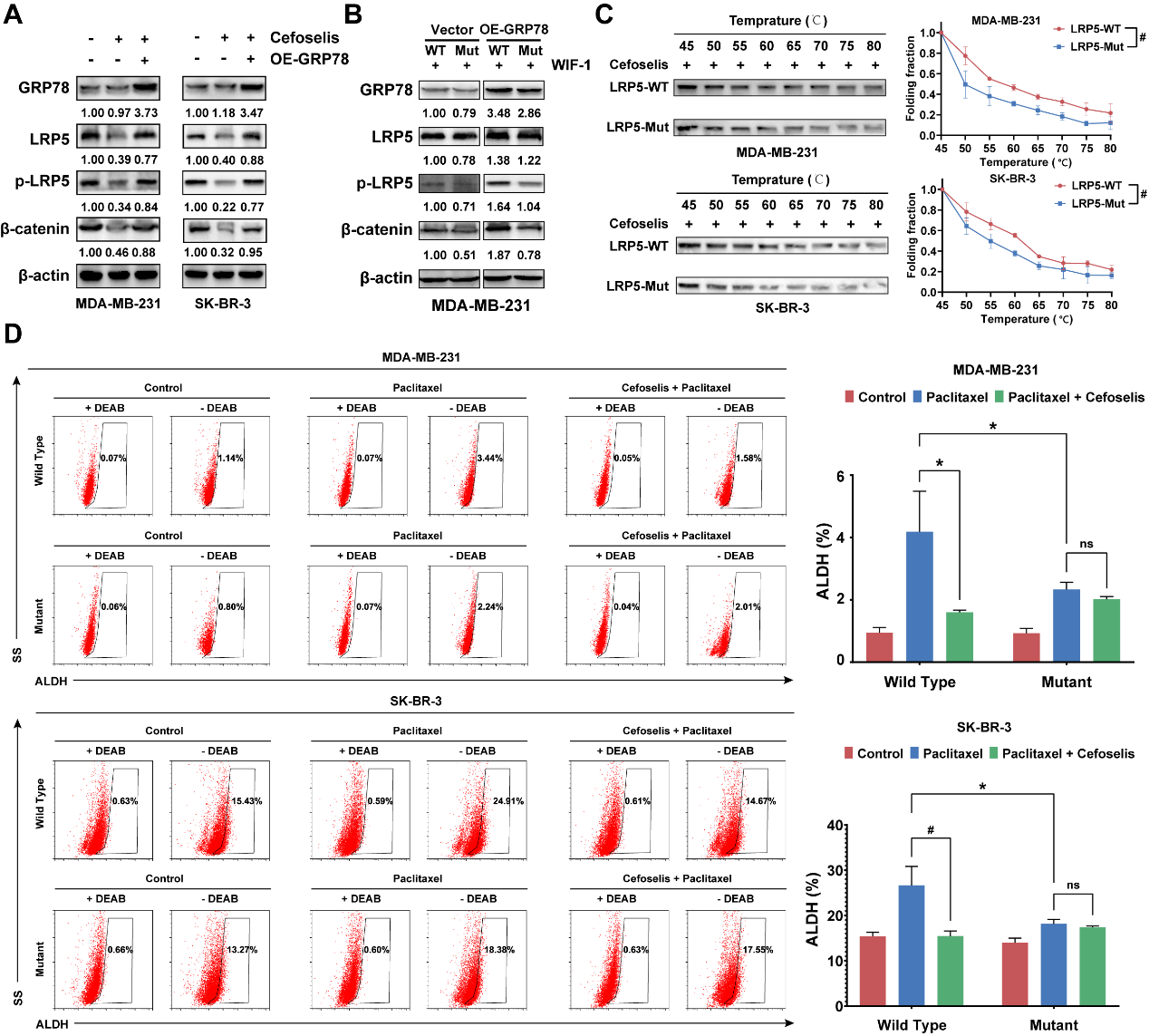
**Figure S9 The PHE294 of LRP5 is a key residue for cefoselis regulating breast cancer cell stemness. (A)** LRP5, p-LRP5, and β-catenin expression in GRP78 overexpressing breast cancer cells were measured following cefoselis (20 μM) treatment. **(B)** The influence of PHE294 mutation in LRP5 on downstream signaling activation was analyzed under the administration of WIF-1 (1 µg/mL) for 24 h. **(C)** The thermal stability of LRP5 protein in breast cancer cells transfected with wild-type LRP5 or PHE294 mutated LRP5 was compared after cefoselis (20 μM) treatment by CETSA assay. **(D)** The effects of cefoselis (20 μM) on paclitaxel-induced (24 nM) ALDH^+^ frequency were detected in the breast cancer cells transfected with wild-type LRP5 or PHE294 mutated LRP5. Data were represented as Mean ± SD. For statistical analysis, ANOVA for repeated measurements (C) and unpaired Student's t-tests (D) were applied. ^*^*P*< 0.05, ^#^*P*< 0.01. OE-GRP78: GRP78 overexpression.
