# Supplementary material for: Cefoselis enhances breast cancer chemosensitivity by directly targeting GRP78/LRP5 signalling of cancer stem cells
Source: Clin Transl Med. 2023 Feb 19;13(2):e1119. doi: 10.1002/ctm2.1119 (PMC9939292; doi:10.1002/ctm2.1119)
Supplement: Supplementary file 3 — Supporting Information [file CTM2-13-e1119-s002.docx]

**Table S1. Correlation between GRP78 expression and clinicopathologic parameters of breast cancer**

| **Parameters** | **Cases** | **GRP78 expression levels** | | ***P* value** | **Correlation Coefficient** |
| --- | --- | --- | --- | --- | --- |
|  |  | **Low** | **High** |  |  |
| **Age** | | | | | |
| ＜50 | 51 | 22 | 29 | 0.333 | 0.090 |
| ≥50 | 67 | 23 | 44 |  |  |
| **Pathological types** |  | | | | |
| Luminal A |  | | | | |
| Yes | 52 | 27 | 25 | 0.001 | -0.322 |
| No | 57 | 12 | 45 |  |  |
| Unclear | 9 | 6 | 3 |  |  |
| Luminal B | | | | | |
| Yes | 20 | 6 | 14 | 0.517 | 0.062 |
| No | 90 | 34 | 56 |  |  |
| Unclear | 8 | 5 | 3 |  |  |
| HER2-overexpressing | | | | | |
| Yes | 16 | 3 | 13 | 0.122 | 0.148 |
| No | 95 | 37 | 58 |  |  |
| Unclear | 7 | 5 | 2 |  |  |
| Triple-negative | | | | | |
| Yes | 21 | 3 | 18 | 0.022 | 0.219 |
| No | 88 | 36 | 52 |  |  |
| Unclear | 9 | 6 | 3 |  |  |
| **Clinical TNM stage** | | | | | |
| T Stage | | | | | |
| T1 | 26 | 15 | 11 | 0.003 | 0.270 |
| T2 | 80 | 29 | 51 |  |  |
| T3 | 12 | 1 | 11 |  |  |
| N Stage | | | | | |
| N0 | 41 | 20 | 21 | 0.039 | 0.191 |
| N1 | 38 | 15 | 23 |  |  |
| N2  N3 | 29 | 8 | 21 |  |  |
|  | 9 | 2 | 7 |  |  |
| Unclear | 1 | 0 | 1 |  |  |

**Spearman rank correlation analysis were performed.**
