# Supplementary material for: Cefoselis enhances breast cancer chemosensitivity by directly targeting GRP78/LRP5 signalling of cancer stem cells
Source: Clin Transl Med. 2023 Feb 19;13(2):e1119. doi: 10.1002/ctm2.1119 (PMC9939292; doi:10.1002/ctm2.1119)
Supplement: Supplementary file 4 — Supporting Information [file CTM2-13-e1119-s001.docx]

**Table S2 Prognostic value of GRP78 for overall survival in breast cancer by univariate and multivariate Cox analysis**

| **Variables** | **Univariate Cox** | | | **Multivariate Cox** | | |
| --- | --- | --- | --- | --- | --- | --- |
|  | **Hazard Ratio** | **95% CI** | ***p*-Value** | **Hazard Ratio** | **95% CI** | ***p*-Value** |
| Age | 1.414 | 0.703-2.842 | 0.331 |  |  |  |
| Luminal A | 0.493 | 0.238-1.024 | 0.058 |  |  |  |
| Luminal B | 0.637 | 0.223-1.817 | 0.399 |  |  |  |
| HER2-overexpressing | 1.791 | 0.776-4.132 | 0.172 |  |  |  |
| Triple-negative | 2.369 | 1.120-5.011 | 0.024 | 1.873 | 0.868-4.038 | 0.110 |
| T Stage | 1.812 | 0.956-3.436 | 0.068 |  |  |  |
| N Stage | 1.52 | 1.080-2.139 | 0.016 | 1.344 | 0.929-4.038 | 0.110 |
| Expression of GRP78 | 2.554 | 1.159-5.629 | 0.020 | 2.341 | 0.930-5.891 | 0.071 |

*P*< 0.05, statistically significant prognostic factor identified by Univariate/Multivariate Cox analysis
