# Supplementary material for: Cefoselis enhances breast cancer chemosensitivity by directly targeting GRP78/LRP5 signalling of cancer stem cells
Source: Clin Transl Med. 2023 Feb 19;13(2):e1119. doi: 10.1002/ctm2.1119 (PMC9939292; doi:10.1002/ctm2.1119)
Supplement: Supplementary file 5 — Supporting Information [file CTM2-13-e1119-s003.docx]

**Table S3. Cefoselis had no significant hepatotoxicity and nephrotoxicity or hematotoxicity *in vivo*.**

| **Blood biochemical values** | **Control** | **Cefoselis (25 mg/kg)** | **Paclitaxel (25 mg/kg)** | **Cefoselis (25 mg/kg)**  **+**  **Paclitaxel (25 mg/kg)** |
| --- | --- | --- | --- | --- |
| ALT (U/L) | 11 ± 4.55 | 11 ± 4.55 | 15.25 ± 9.54 | 11 ± 6.68 |
| AST (U/L) | 86.75 ± 32.29 | 68.25 ± 25.3 | 65 ± 27.83 | 70.25 ± 33.27 |
| Urea (mmol/L) | 3.05 ± 0.7 | 2.05 ± 0.19 | 2.63 ± 0.86 | 2.58 ± 0.19 |
| Cr (μmol/L) | <5 | <5 | <5 | <5 |
| UA (μmol/L) | 58.25 ± 24.09 | 69.75 ± 38.67 | 75 ± 47.97 | 94 ± 28.93 |
| WBC (10^9^/L) | 323.97 ± 42.44 | 303.06 ± 8.37 | 183.79 ± 44.99* | 195.86 ± 35.62* |
| RBC (10^12^/L) | 9.32 ± 0.64 | 9.19 ± 1.29 | 8.29 ± 1.36 | 7.88 ± 1.72 |
| Hb (g/L) | 134.75 ± 9.11 | 131.75 ± 24.69 | 119 ± 20.58 | 117.25 ± 17.48 |

**ALT, Alanine transaminase; AST, Aspartate aminotransferase; Cr, Creatinine; UA, Uric acid; WBC, White blood cell; RBC, Red blood cell; Hb, Hemoglobin. Data were represented as mean values ± SD, **P* <0.05 *vs* Control.**
